# SREBP-2-driven transcriptional activation of human SND1 oncogene

**DOI:** 10.18632/oncotarget.22569

**Published:** 2017-11-21

**Authors:** Sandra Armengol, Enara Arretxe, Leire Enzunza, Irati Llorente, Unai Mendibil, Hiart Navarro-Imaz, Begoña Ochoa, Yolanda Chico, María José Martínez

**Affiliations:** ^1^ Lipids & Liver Research Group, Department of Physiology, Faculty of Medicine and Nursing, University of the Basque Country UPV/EHU, Barrio Sarriena s/n, 48940 Leioa, Vizcaya, Spain

**Keywords:** SND1, Tudor-SN, gene promoter regulation, SREBP-2, SREBP-1

## Abstract

Upregulation of Staphylococcal nuclease and tudor domain containing 1 (SND1) is linked to cancer progression and metastatic spread. Increasing evidence indicates that SND1 plays a role in lipid homeostasis. Recently, it has been shown that SND1-overexpressing hepatocellular carcinoma cells present an increased *de novo* cholesterol synthesis and cholesteryl ester accumulation. Here we reveal that SND1 oncogene is a novel target for SREBPs. Exposure of HepG2 cells to the cholesterol-lowering drug simvastatin or to a lipoprotein-deficient medium triggers SREBP-2 activation and increases SND1 promoter activity and transcript levels. Similar increases in SND1 promoter activity and mRNA are mimicked by overexpressing nuclear SREBP-2 through expression vector transfection. Conversely, SREBP-2 suppression with specific siRNA or the addition of cholesterol/25-hydroxycholesterol to cell culture medium reduces transcriptional activity of SND1 promoter and SND1 mRNA abundance. Chromatin immunoprecipitation assays and site-directed mutagenesis show that SREBP-2 binds to the SND1 proximal promoter in a region containing one SRE and one E-box motif which are critical for maximal transcriptional activity under basal conditions. SREBP-1, in contrast, binds exclusively to the SRE element. Remarkably, while ectopic expression of SREBP-1c or -1a reduces SND1 promoter activity, knocking-down of SREBP-1 enhances SND1 mRNA and protein levels but failed to affect SND1 promoter activity. These findings reveal that SREBP-2 and SREBP-1 bind to specific sites in SND1 promoter and regulate SND1 transcription in opposite ways; it is induced by SREBP-2 activating conditions and repressed by SREBP-1 overexpression. We anticipate the contribution of a SREBPs/SND1 pathway to lipid metabolism reprogramming of human hepatoma cells.

## INTRODUCTION

Staphylococcal nuclease and tudor domain containing 1 gene (*SND1*) encodes the conserved multidomain protein SND1, also known as Tudor-SN, TSN or p100 [1–4]. SND1 has been reported to orchestrate multiple functions in the regulation of gene expression, including transcriptional activation [5–8], spliceosome assembly and pre-mRNA splicing [9, 10], RNA interference, stability and editing [11, 12], RNA protection in stress granules [13, 14], and regulation of protein synthesis, ubiquitination and proteasomal degradation [15, 16].

Emerging findings have demonstrated that SND1 overexpression is linked to progression and malignancy of various types of cancer, such as colon, breast, prostate, lung, glioma, melanoma and liver cancer [17–23]. These studies documented multiple ways for SND1 to facilitate carcinogenesis. Participation of SND1 in molecular networks involving NF-κB signalling activation and miR-221 induction [23], miR-184 expression and JAK/STAT3 inhibition [21], TGFβ1/Smad signalling pathway [15], Wnt/β-catenin activation [17] as well as the interaction of SND1 with partner proteins like metadherin-1 [24] and monoglyceride lipase [16], have been described to strictly modulate prosurvival and proliferative genes and proteins expression in cancer cells. The tumour type-selective oncogenic functions assigned to SND1 may be regarded as a result of the ubiquitous expression of the protein [25] and its ample capacity for interacting with nucleic acids and proteins in the nuclear [26] and extranuclear compartments [27–29]. It is in that context where our recent studies in human hepatoma HepG2 cells demonstrated the interaction of nuclear SND1 with the genomic DNA and the recruitment of SND1 to the promoter of a wide number of target genes modulating cell growth, oncogenic transformation, viral infection and metabolic regulation [30].

A reprogrammed lipid metabolism and a lipogenic phenotype are features that distinguish cancer cells from normal cells [31]. Lipids are key metabolic substrates for providing energy and building units for the newly forming cells; but, they also generate a network of protumorigenic signals that promote cancer growth [31]. However, the precise mechanisms through which oncogenes alter lipid metabolism are yet poorly defined. There is increasing evidence indicating that SND1 plays a role in specific aspects of lipid bodies biogenesis and the secretion of lipid in the milk by mammary epithelial cells [27, 32] or in the lipoprotein particles by liver cells [29, 33]. Work from our group in knocking-down experiments demonstrated for the first time a role for SND1 in the control of expression of genes regulating glycerophospholipid homeostasis and phosphatidylcholine content during the inflammatory response [30]. A very recent insight associated the overexpression of SND1 with an enhanced cholesterol biosynthesis and storage of cholesteryl esters in rat hepatoma cells, suggesting that SND1 may be decisive to determine events that modify the permeability properties of cancer cell membranes and facilitate cell proliferation [34]. Despite all these efforts, the precise function of SND1 in managing lipid metabolism in proliferating cells and the molecular mechanisms regulating SND1 gene expression are important questions that remain to be fully understood.

Mammalian sterol regulatory element binding proteins (SREBPs) are master regulators of sterol and fatty acid homeostasis [35–37]. The three isoforms of SREBPs are encoded by two genes: *SREBF1* originates SREBP-1a and SREBP-1c and a separated gene *SREBF2* encodes SREBP-2. Although significant functional overlap between SREBPs exists, SREBP-1c is the main responsible for activation of genes involved in fatty acid, phospholipid and triacylglycerol synthesis, SREBP-2 primarily governs cholesterol synthesis and uptake while SREBP-1a can regulate both pathways. However, the three SREBPs differ in their tissue distribution and responses to regulatory challenges. SREBP-1c and SREBP-2 are the predominant isoforms expressed in mammalian liver although HepG2 and other types of cultured cells produced predominantly SREBP-1a and SREBP-2 [38, 39]. SREBPs are synthesized as inactive precursors bound to the endoplasmic reticulum (ER) membrane and are subjected to complex posttranslational regulation. When the sterol concentration in ER membranes is high, SREBPs are retained in the membrane in association with SREBP cleavage activating protein (SCAP) and insulin induced gene protein (Insig), that are sensitive to ER membrane sterol levels. In response to low sterol levels, the SCAP/SREBP complex is released from Insig and escorted to Golgi where SREBP undergoes proteolytic cleavage. The released active amino-terminal fragment translocates to the nucleus and binds to sterol response elements (SRE) or to palindromic E-boxes and transactivates target genes [35, 40]. SREBPs are relatively weak activators of transcription, and for maximal action they commonly require cooperation with one or more accessory transcription factors, most commonly including Sp1 (specificity protein 1), NF-Y (nuclear factor Y) or both [41].

Our previous studies provided the characterization of the human SND1 gene promoter (GenBank ID: EF690304). It is a TATA-less promoter containing conserved CCAAT and GC boxes for the functional binding of NF-Y and Sp1 transcription factors as well as several NF-κB binding sites that play a regulatory role in the TNFα-induced activation of SND1 transcription [42–44] and a number of ER stress response elements [45]. In addition, the existence of sterol response elements within SND1 promoter has been predicted *in silico*. Here we reveal that SREBP-2 and SREBP-1 bind to specific sites in the promoter region and regulate SND1 transcription in opposite ways. Binding sites for SREBPs have been analysed *in vivo* and *in vitro* and their role on SND1 promoter was investigated by determining the transcriptional activity of functional and mutated 5’-deletion fragments. Our findings uncover SND1 as a novel target gene for SREBPs that is induced by SREBP-2 activation upon conditions of sterol depletion in the hepatoma cell and repressed by SREBP-1.

## RESULTS

### Response of SND1 expression to SREBP-2 activity modulators

**Figure 1:**
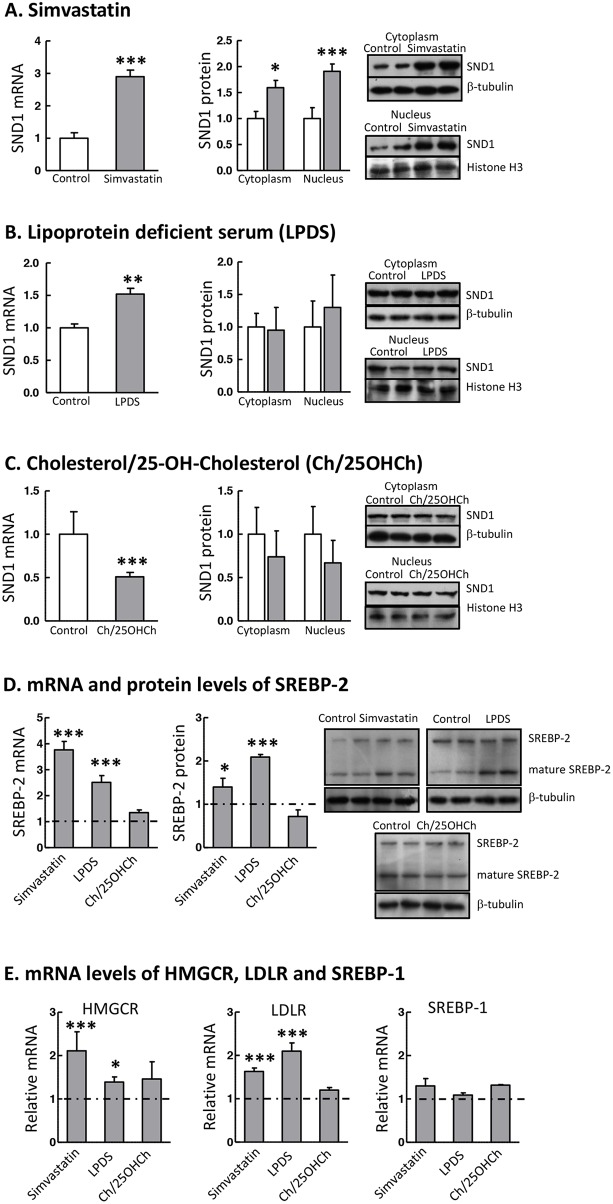
SND1 gene transcription responds to SREBP-2 activity modulators The SND1 transcript level and the SND1 protein content in nuclei and the cytoplasmic fraction were quantified in control HepG2 cells (white bars) and cells cultured during 24 h with 10 μM simvastatin **(A)**, or in a lipoprotein deficient (LPDS) medium **(B)**, or in the presence of 10 μg/ml cholesterol plus 1 μg/ml 25-hydroxycholesterol (Ch/25OHCh) **(C)** (dark bars). Aliquots of cells (7 x10^5^ cells) were subjected to RNA isolation and first strand cDNA was synthesized and used as the template for individual PCR reactions using the primers listed in Supplementary Table 1. Other aliquots (7 x10^5^ cells) were processed for the isolation of nucleus and cytoplasm and subjected to immunoblot analysis for SND1 and normalized with histone H3 and β-tubulin, respectively. The precursor (SREBP-2) and mature SREBP-2 protein content **(D)** and the HMGCR, LDLR and SREBP-1 transcript levels **(E)** were also determined in cells treated as above, and are expressed relative to the level in control cells, which is shown as a grey grid line. Results are reported as the mean ± SD of 3-5 independent experiments, each performed in triplicate (duplicate in western blotting), and were analyzed by the two-tailed Student's *t*-test. ^*^ p≤0.05, ^**^ p≤0.01 and ^***^ p≤0.001 denote the effect of treatment.

To determine whether SND1 expression was regulated by SREBP-2, human HepG2 cells were cultured under different cellular conditions that modulate SREBP-2 pathway. Sterol starvation triggers the activation of SREBPs pathway and the cleavage of SREBP-2 to the active form. Thus, cells were treated with the cholesterol lowering drug simvastatin or were grown in a culture medium with lipoprotein deficient serum (LPDS) in order to activate SREBP-2. Simvastatin is an inhibitor of hydroxymethylglutaryl-coenzyme A reductase (HMGCR), the enzyme catalysing the rate limiting step in cholesterol biosynthesis, whereby the mevalonate pathway is inhibited and the intracellular cholesterol levels diminish. We confirmed that HepG2 treatment with 10 μM simvastatin or the cell culture in LPDS medium resulted in the induction of SREBP-2 mRNA and protein expression, concomitant with the increase of transcripts of downstream target genes HMGCR and LDL receptor (LDLR) and unchanged SREBP-1 mRNA content (Figure 1D and 1E). In parallel, we found that simvastatin or LPDS significantly up-regulated (3-fold or 1.6-fold) the expression of SND1 mRNA in human hepatoma cells and that SND1 protein accumulated in both the nucleus and the cytoplasm of simvastatin-treated cells (Figure 1A), though it was not significantly altered by LPDS (Figure 1B).

To asses the response of SND1 gene expression to a sterol rich condition, HepG2 cells were incubated during 24 h in the presence of 10 μg/ml cholesterol plus 1 μg/ml 25-hydroxycholesterol. These sterols bind to SCAP and Insig, respectively, impairing the ER-to-Golgi transfer of SREBPs and their travelling to the nucleus, thus inhibiting SREBP-2 and SREBP-1 pathways. As shown in Figure 1C, a significant decrease of about 50% was noticed in the SND1 transcript level, although SND1 protein remained invariable. The transcript level of expression of SREBP-2 and its target genes was unmodified by sterols (Figure 1D and 1E).

**Figure 2:**
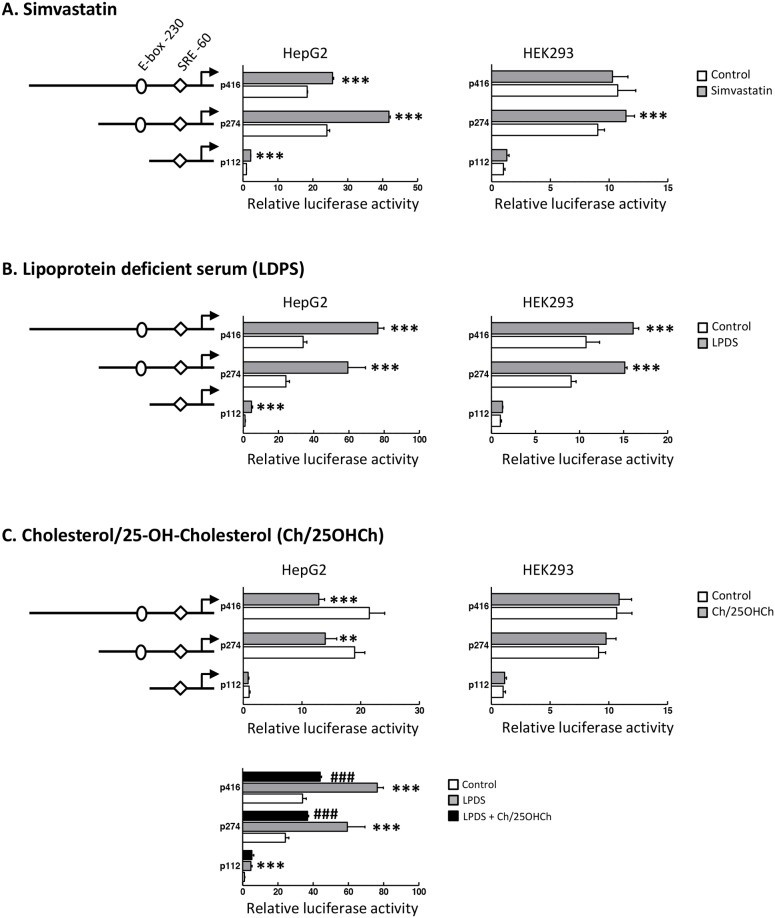
Transcriptional activity of SND1 proximal promoter responds to SREBP-2 activity modulators SND1 transcriptional activity was measured in HepG2 and HEK293 cells (10 x10^3^) transiently transfected with SND1 promoter constructs p112, p274 or p416 covering the promoter sequence (-112, +221) or (-274, +221) or (-416, +221) cloned in pGL3-Basic vector as described in Material and methods and cultured during 24 h with 10 μM simvastatin **(A)**, or in a lipoprotein deficient (LPDS) medium **(B)**, or in the presence of 10 μg/ml cholesterol plus 1 μg/ml 25-hydroxycholesterol (Ch/25OHCh) **(C)** (dark bars). Promoter activity is represented as luciferase arbitrary units relative to its corresponding control, untreated p112 fragment. Results are reported as the mean ± SD of 3-5 independent experiments, each performed in quadruplicate and were analyzed by the two-tailed Student's *t*-test. ^**^ p≤0.01 and ^***^ p≤0.001 denote the effect of treatment, ^###^p≤0.001 denotes the effect of Ch/25OHCh+LPDS versus LPDS alone.

Next, we investigated whether the SND1 promoter transcriptional activity changed accordingly to the changes in SND1 mRNA expression and SREBP-2 activity. We assayed HepG2 cells transfected with 5’-deletion fragments of the SND1 promoter in the presence or absence of simvastatin, LPDS or exogenous sterols. We firstly constructed plasmids that contained SND1 promoter sequences covering the regions from nucleotide -112 or -274 or -416 to +221 (relative to the transcriptional start site) ahead from the luciferase coding region into pGL3-Basic as detailed in previous work [44]. A consistent increase in the reporter activity of the SND1 deletion fragments was observed in the transfected HepG2 cells following exposure to simvastatin (Figure 2A) or LPDS medium (Figure 2B). Conversely, exogenous sterols not only reduced luciferase activity (Figure 2C, upper panel) but also counteracted the LPDS-mediated activation of SND1 promoter activity (Figure 2C, lower panel). Transfection assays of human embryonic kidney HEK293 cells, used as a non-hepatic non-tumoral cell line model, exhibited similar SND1 promoter activation by simvastatin and LPDS, with the exception of the lack of response of fragment 416 to simvastatin (Figure 2A and 2B). Also of note is the absence of promoter response to exogenous sterols in HEK293 cells (Figure 2C). Altogether these results are consistent with the concept that there is a sterol-sensitive mechanism of transcriptional regulation operating for the SND1 gene in human hepatoma cells which seems to be somewhat less operative in HEK293 cells. Both, the activation of the SND1 proximal promoter and the upregulation of SND1 expression upon sterol deprivation, point to SND1 as a novel SREBP-2 inducible gene.

### SND1 promoter contains functional binding sites for SREBPs

**Figure 3:**
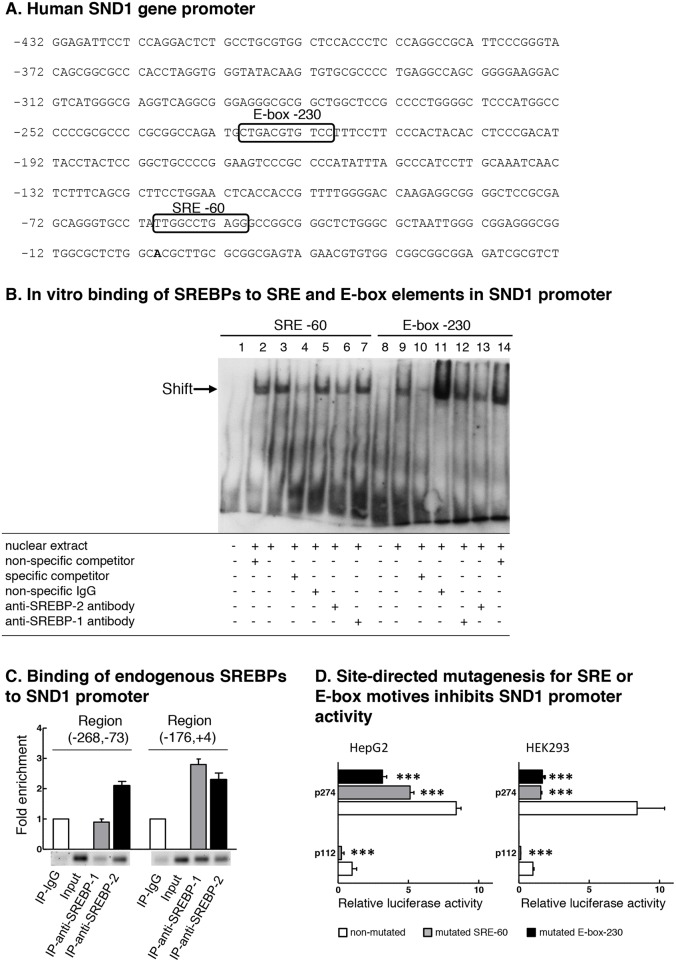
SND1 promoter contains functional binding sites for SREBPs **(A)** Partial nucleotide sequence of SND1 gene proximal promoter [GenBank: EF690304]. The transcription start site (+1) is shown in bold. Boxes indicate predicted binding motives for SREBP transcription factors SRE -60 and E-box -230. **(B)** Electrophoretic mobility shift assay for the predicted SRE and E-box sequences using HepG2 nuclear extracts. Competition assays were performed with 100x excess of specific or non-specific unlabelled probe. Lanes 1 and 8 free probe, lanes 3 and 9 HepG2 nuclear extracts, lanes 2 and 14 non-specific competitor, lanes 4 and 10 SRE -60 and E-box -230 specific competitor, respectively, lanes 5 and 11 non-specific antibodies, lanes 6 and 13 anti-SREBP-2 IgG, and lanes 7 and 12 anti-SREBP-1 IgG. **(C)** Binding of endogenous SREBP-1 and SREBP-2 to SND1 gene promoter. Chromatin from HepG2 cells was immunoprecipitated with anti-SREBP-2, anti-SREBP-1, or non-immune IgG as negative control. DNA from input or immunoprecipitates (IP) was subjected to PCR to amplify SND1 promoter (-268, -73) and (-176, +4) regions containing the predicted SRE or E-box elements. Results in B and C are representative of three experiments with similar results. **(D)** Site-directed mutagenesis for SRE -60 or E-box -230 motif was performed in HepG2 and HEK293 cells as described in Material and methods and SND1 promoter activity measured and expressed relative to non-mutated p112 luciferase activity. Results are reported as the mean ± SD of 3 independent experiments, each performed in quadruplicate, and were analyzed by the two-tailed Student's *t*-test. Significance is denoted: ^***^ p≤0.001.

In order to address the binding of SREBP-2 transcription factor to the SND1 promoter, we first sought for potential binding sites within the promoter sequence using MatInspector [46] and Jaspar [47] bioinformatics tools. Figure 3A shows the sequence of SND1 proximal promoter with the predicted sterol response element similar to classic SRE and the E-box found by both programs at positions -60 and -230 upstream the transcription start site, respectively. There are also four potential regulatory elements in the distal promoter: two SRE motives at -772 and -1092 and two E-box elements at -934 and -1237 (Supplementary Figure 1).

We explored the *in vitro* binding of SREBP-2 and SREBP-1 to the SRE -60 and the E-box -230 regulatory elements and performed EMSA experiments using nuclear extracts from HepG2 cells and digoxigenin-labelled oligonucleotides containing the sequences of SRE -60 (Figure 3B, lanes 1-7) and the canonical E-box -230 site (Figure 3B, lanes 8-14) of the SND1 promoter. Figure 3B shows the formation of two specific DNA-protein complexes, one with SRE (lane 3) and one with E-box (lane 9) sequence. The specific binding was abolished by an excess of the specific unlabelled probe (lanes 4 and 10) but not by the non-specific probe for Oct2A (lanes 2 and 14) or by non-specific IgG (lanes 5 and 11). Although the specific bands were not supershifted by anti-SREBP-1 (lanes 7 and 12) or anti-SREBP-2 (lanes 6 and 13) antibodies, we observed that the anti-SREBP-2 IgG and not anti-SREBP-1 IgG weakened both the SRE-DNA and the E-box-DNA interactions, suggesting SREBP-2 binding to the SRE and E-box probes.

Chromatin immunoprecipitation (ChIP) assays were performed to verify the binding of endogenous SREBPs to the SND1 gene promoter in HepG2 cells. After cross linking, chromatin was immunoprecipitated with specific antibodies directed against SREBP-2 or SREBP-1 or non-immune IgG (negative control). Then, the regions (-176, +4) and (-268, -73) of SND1 gene promoter containing the potential SREBP binding sites were amplified from the immunoprecipitates. As Figure 3C shows, both SREBP-1 and SREBP-2 bound to elements within the promoter region (-176, +4) where SRE -60 site is located and a 2.4-3-fold enrichment for the binding sites was rendered for each amplified DNA. Amplification of the region (-268, -73) containing the predicted E-box -230 showed the association of SREBP-2 (2.2-fold enrichment) but not of SREBP-1 with the regulatory elements in this promoter region, revealing that the SREBP-2 and SREBP-1 transcriptional factors occupy some specific sites in SND1 proximal promoter.

The functional role of these regulatory elements was demonstrated by mutational analysis of the SND1 promoter. Site-directed mutagenesis to the core sequence for the SRE -60 or the E-box -230 motif was performed in the 5’-deletion fragments p112 and p274. The reporter constructs carrying the individual SRE or E-box mutations rendered a reduced luciferase activity (40-70%) respect to that measured in the wild-type constructs in HepG2 cells (Figure 3D). A more dramatic inhibition (80-90%) in the luciferase activity of mutated fragments was measured in HEK293 cells (Figure 3D). Such reductions in transcriptional activity strongly suggest that these SREBP binding sites are regulatory elements for the promoter function and transcription of the human SND1 gene.

### SND1 expression positively correlates with the expression level of SREBP-2

**Figure 4:**
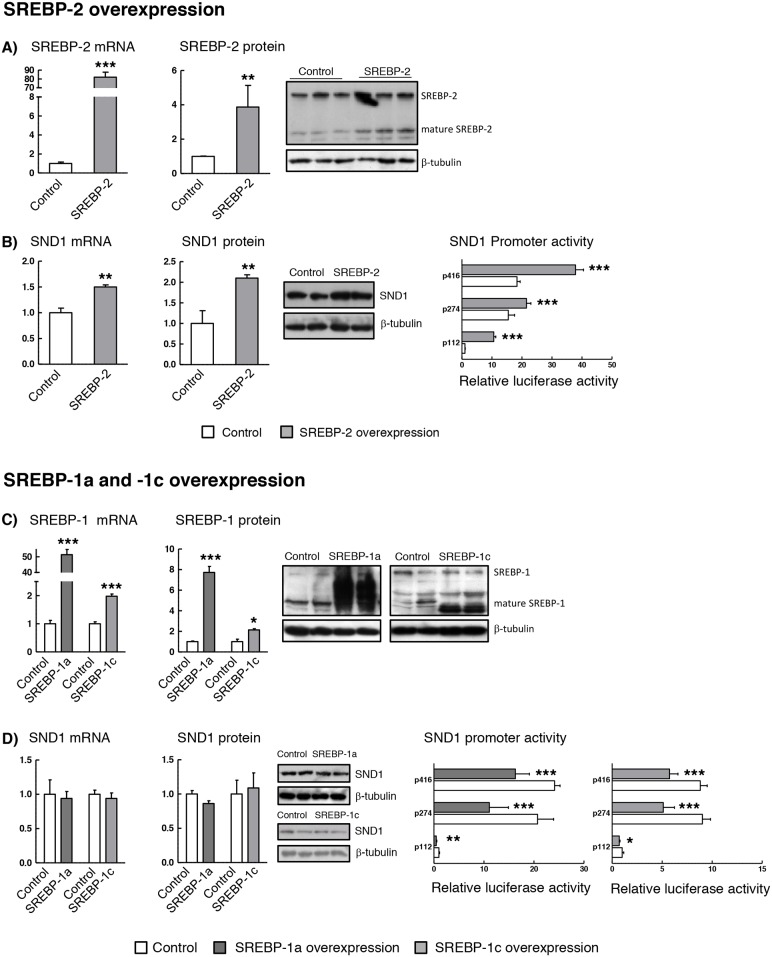
SND1 gene transcription is dependent on the cellular SREBP levels HepG2 cells were transfected with expression plasmids of human SREBP-2 **(A and B)** or SREBP-1a or SREBP-1c **(C and D)**. After 24h, (A and C) SREBPs and (B and D) SND1 transcript and protein levels and SND1 gene promoter activity were determined as described in Material and methods. Transcript and protein levels are expressed relative to the level in control cells. Promoter activity is expressed as luciferase arbitrary units relative to its corresponding p112 control. Results are reported as the mean ± SD of 3 independent experiments, each performed in quadruplicate (triplicate or duplicate in western blotting), and were analyzed by the two-tailed Student's *t*-test. Significance is denoted: ^*^ p≤0.05, ^**^ p≤0.01 and ^***^ p≤0.001 versus control cells.

In order to delineate the regulatory role of SREBPs on SND1 gene expression, we performed gain-of-function and loss-of function experiments. Transient cotransfection of HepG2 cells with SND1 promoter constructs plus human SREBP-2 or SREBP-1a or -1c expression plasmids were carried out. Cotransfected cells displayed a very efficient mRNA overexpression of SREBP-2 (80-fold) (Figure 4A) and SREBP-1a (50-fold) and a more modest increase in the SREBP-1c mRNA (2-fold) (Figure 4C). In general, a less strong effect on the corresponding mature protein (2-8 fold) was detected by Western blotting (Figure 4A and 4C). SREBP-2 overexpression resulted in significant activation of SND1 promoter activity, which was accompanied by a rise in SND1 mRNA and protein (Figure 4B), the latter being mainly accumulated in the nucleus (data not shown). On the contrary, and remarkably, cells overexpressing SREBP-1a or SREBP-1c displayed a marked decrease in the SND1 transcriptional activity and no alterations in SND1 transcript and protein amount (Figure 4D). Such contrasting effects of SREBP-2 and SREBP-1a and -1c on SND1 gene promoter transcriptional rate point to a dual regulatory mechanism for SND1 expression in hepatoma cells.

**Figure 5:**
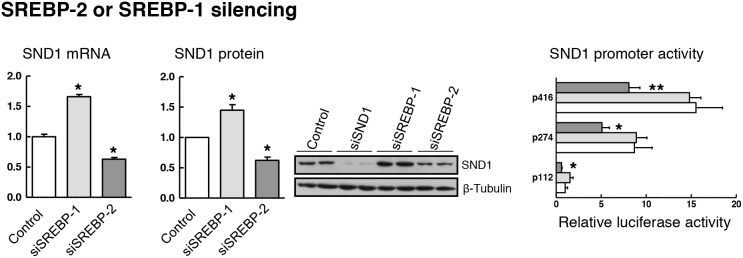
SREBP-1 or SREBP-2 depletion affects SND1 gene transcription For knocking-down assays, reverse transfection was performed with 10 nM specific siRNAs for SREBP-1 or SREBP-2, and SND1 mRNA, protein and luciferase activity were determined in HepG2 cells expressing either basal (white bars) or residual levels of SREBPs after silencing endogenous SREBP-1 (light grey) or SREBP-2 (dark grey). Transcript and protein levels are expressed relative to the level in control cells. Silencing efficiency was monitored through the residual SND1 protein detected after 10 nM siSND1 transfection, and promoter activity is expressed as luciferase arbitrary units relative to its corresponding p112 control. Results are reported as the mean ± SD of 3 independent experiments, each performed in triplicate (duplicate in western blotting), and were analyzed by the two-tailed Student's *t*-test. Significance is denoted: ^*^ p≤0.05 and ^**^ p≤0.01 versus control cells.

Next, we evaluated SND1 gene expression in HepG2 cells with siRNA-mediated depletion of SREBP-2 or SREBP-1. The deletion was induced by transfecting cells with siRNAs directed against regions 1193-1211 or 1566-1584 of SREBP-2 mRNA or regions 505-523 or 4044-4062 of SREBP-1 transcript. The silencing of each SREBP was successful as evidenced by the residual amount of SREBP-2 and SREBP-1 mRNA and the reduced nuclear protein detected in the siRNA-transfected cells compared to the mock-transfected cells (Supplementary Figures 2 and 3). No cross-reactions between the siRNAs for SREBP-1 and SREBP-2 transcript were confirmed, but we discarded the SREBP-2 siRNA matching region 1193-1211 because it cross-reacted with SREBP-1 gene and reduced by about 50% the expression of SREBP-1 transcript. Ten nM siRNAs and 48 h were defined as optimal parameters for HepG2 cells considering the maximal inhibition of the SREBP-2 target gene HMGCR transcript and the marked decrease in free and esterified cholesterol content (Supplementary Figure 2C and 2D), and the impaired HepG2 cell proliferation due to SREBP-2 deletion from 48 h ahead (Supplementary Figure 2E). Inhibition of endogenous SREBP-2 by siRNA reduced the activity of SND1 promoter and the expression of SND1 mRNA and protein (Figure 5). However, SREBP-1 silencing did not affect luciferase activity of the SND1 5’-deletion fragments although it augmented the level of expression of SND1 transcript and protein (Figure 5).

Collectively, these findings evidence that SND1 transcription is controlled by SREBPs and the binding of SREBP-2 and/or SREBP-1 to specific sites in the gene promoter might ensue opposite responses of SND1 gene expression, at least in the hepatic HepG2 cell context.

## DISCUSSION

Here we attempt to decipher the transcriptional control of SND1 gene by SREBP-2 and SREBP-1 transcription factors in human hepatoma cells. The first novel result in the present study is a neat SREBP-2-mediated activation of SND1 expression under cholesterol-lowering conditions. This is the classic model of regulation of SREBPs activity by intracellular sterol concentration that fits well with SREBP-2. Lipoprotein deficiency or simvastatin treatment activated SREBP-2 and raised SND1 promoter activity and mRNA abundance while the addition of sterols to the culture medium decreased SND1 promoter activity and mRNA expression. The mixture of cholesterol and 25-hydroxycholesterol seemed to be insufficient to affect the regulatory pool of cholesterol and did not produce consistent reduction in SREBP-2 transcript and nuclear protein (Figures 1 and 2). However, taking into account that 25-hydroxycholesterol belongs to a large family of oxysterols that can bind different nuclear receptors, such as liver X receptor (LXR), and/or other regulatory proteins [48, 49] and may serve as important modulator of the expression of multiple genes, it is tempting to speculate that SND1 could be included among them. The SREBP-2 gain-of-function and loss-of function experiments reinforced the positive connection between SND1 promoter activity and transcript expression, and SREBP-2 activity (Figures 4 and 5). Moreover, ChIP assays confirmed the binding of endogenous SREBP-2 to two SND1 promoter regions exhibiting recognition domains, SRE -60 and E-box -230, and mutational analysis demonstrated the functional role for these two binding sites in the positive regulation of the human SND1 promoter activity by SREBP-2 transcription factor (Figure 3). A similar regulatory mechanism seems to operate over SND1 promoter in HEK293 cells with a few divergences in the responsiveness to sterol (Figures 2 and 3) that may be attributed to a different network of transcription factors operating in these non-hepatic, non-tumoral cells compared to HepG2 cells.

Our study also reveals that endogenous SREBP-1 occupies a specific site in the promoter region containing the regulatory element SRE -60, and that, SREBP-1a or SREBP-1c overexpression led to an unexpected reduction in the transcriptional activity of SND1 promoter that did not affect the level of SND1 transcript (Figure 4). It is interesting to mention that knocking down SREBP-1 released SND1 promoter from the SREBP-1 repressive action and raised the cellular amount of SND1 mRNA and protein. This observation leads us to suggest that SREBP-1 either serve as an SND1 promoter inhibitor or affect other posttranscriptional processes regulating SND1 expression.

Opposite effects of SREBPs have been described for a few genes [50]. In light of our findings, we can speculate that SREBP-2 or SREBP-1 isoforms bind the SRE -60 site in SND1 promoter activating or inactivating its transcriptional activity in response to upstream signals and the set of transcription factors and cofactors recruited to the gene promoter. Overlapping between SREBP-2 and SREBP-1 target genes has been previously reported in liver cells even though each isoform appears to bind to different set of targets depending on the particular cellular environment [53, 54] and the structure of the gene promoter [39]. Differential transactivity of SREBP-1a, -1c and -2 on SREBP-target promoters is defined by the position of their SRE and E-box motifs and the requirement of other transcription factors as cofactors for the SREBPs activation. It is well established that SREBPs usually cooperate with transcription factors such as Sp1 and NF-Y to exert their regulatory effects on their target genes [41, 55] and that nucleotide spacing length between the SRE and NF-Y or Sp1 binding sites is decisive for optimal sterol-dependent transcriptional regulation [55]. The SND1 gene promoter contains conserved Sp1 and NF-Y sites that are located close to or even overlap the SREBPs recognition domain SRE -60 [44]. Preliminary findings point to that SREBP-2-mediated stimulation of SND1 promoter activity is independent on the binding of Sp1 and/or NF-Y to the promoter and a combination with NF-Y is required for the efficient inhibition of SND1 promoter activity by SREBP-1 (Supplementary Figure 4). We have to keep in mind that both several upstream SRE and/or E-box elements in the distal promoter (Supplementary Figure 1) and the regulatory network of proteins interacting with the SND1 promoter sequence (Supplementary Figure 5) could play relevant roles in the regulation of SND1 gene expression by SREBPs. Because of the complex networks that control SREBPs functions [37, 51, 52], further studies should be carried out to understand the precise molecular mechanism and the combination of transcription factors required in the control of SND1 promoter by SREBP transcription factors.

Besides the transcriptional control of SND1 gene, other alternative effects of SREBPs may occur at the postranscriptional level and affect the SND1 mRNA processing and the formation of the protein. Both *SREBF2* and *SREBF1* encode microRNAs, miR-33a and miR-33b respectively, and the coordinate expression of the corresponding SREBP/miRNA represents a mechanism to regulate the expression of the genes responsible of cholesterol homeostasis maintenance [56]. Whether SND1 gene might be included within this group is an intriguing hypothesis to be evaluated. Up to date, the limited information relative to the postranscriptional regulation of SND1 identifies SND1 transcript as a target of miR-184 in malignant gliomas and breast cancer [21, 57] and miR-361-5p in colorectal and gastric cancer [58] and links miRNAs suppression with SND1 upregulation and cancer development and progression.

Our findings here reported about the SREBP-2-mediated activation of SND1 transcription in response to sterol-lowering treatments are consistent with a recent study in rat hepatoma cells overexpressing SND1 that evidence a constitutive overactivation of the SREBP-2 regulatory system [34]. The authors documented that SND1 overexpression deregulated cholesterol metabolism and increased the content of free and esterified cholesterol; despite the high intracellular cholesterol level, regulatory cholesterol pools in the ER membranes were altered and SREBP-2 proteolytic activation was favoured [34]. We assume as a hypothesis a regulatory feed-forward loop between SREBP-2 and SND1 transcription and consequently, the overexpression of SND1 may disrupt cholesterol homeostasis and activate the SREBP-2 pathway in the hepatic cancer cells.

In summary, this study provides first evidence to support that human SND1 oncogene is under the control of SREBP-2 and SREBP-1 and points to a novel SREBPs/SND1 pathway that highlights the necessity for defining their upstream modulating signals and their downstream effector molecules to better understand the relative contribution of SND1 to lipid metabolism in liver cancer cells.

## MATERIALS AND METHODS

### Cell culture and treatments

Human hepatocellular carcinoma HepG2 cells (ATCC) and human embryonic kidney HEK293 cells (ATCC) were grown in EMEM (ATCC) supplemented with 2 mM L-glutamine, 100 U/ml penicillin, 100 μg/ml streptomycin (Sigma-Aldrich) and 10% (v/v) foetal bovine serum (ATCC) at 37°C and 5% CO_2_. In some experiments foetal bovine serum was replaced by 10% lipoprotein deficient serum (LPDS) (Sigma). For RNA or protein determination 7×10^5^ cells were seeded in 6 well plates; and for measurement of SND1 transcriptional activity 10×10^3^ cells/well in 96 well plates were used. When indicated, cells were treated for 24 h with 10 μM simvastatin or with a mixture of 10 μg/ml cholesterol plus 1 μg/ml 25-hydroxycholesterol; same volume of solvent, DMSO or ethanol respectively, was added to control cells.

### Transient transfection and luciferase reported assay

HepG2 cells were transiently transfected with 0.1 μg SND1 constructs p112, p274 and p416, which cover the promoter regions (-112, +221), (-274, +221) and (-416, +221) cloned into the Firefly luciferase reporter vector pGL3-Basic (Promega) and 0.1 μg of Renilla luciferase pRL-TK (Promega), using XTreme9 transfection reagent (Roche Applied Science) as described in [44]. Mutants of constructs p112 and p274 containing mutations in SRE (TctaCCatcaG, the underlined sequences indicate the mutated nucleotides) or E-box (CTccgtgaTCC) element were cloned into the Firefly luciferase vector pRP (VectorBuilder, Cyagen Biosciences). HEK293 cells were transfected with 0.05 μg reporter gene constructs plus 0.05 μg Renilla luciferase. After 24 h, cells were lysed and luciferase activity was determined in quadruplicate from promoter constructs by the Dual-Luciferase Reporter Assay System (Promega). *Firefly luciferase* activity was normalized to *Renilla luciferase*. Luciferase data were expressed as relative luminescence units (RLU), setting to 1.0 the value for p112 fragment.

In the transactivation experiments cells were cotransfected with 0.1 μg pcDNA3-SREBP-1a plasmid (gift from Timothy Osborne, Addgene plasmid # 26801) or pSVSport-SREBP-1c plasmid (gift from Bruce Spiegelman, Addgene plasmid # 26807) or pcDNA3-SREBP-2 (gift from Timothy Osborne, Addgene plasmid # 8883). As negative controls the empty pCDNA3 vector or the pSVSport SREBP-1c dom neg, which expresses a non-functional mature form of SREBP-1c substituying Tyr-320 by Ala (gift from Bruce Spiegelman, Addgene plasmid # 8885), were used. In these experiments pSV-β-Galactosidase was used for controlling transfection efficiency and *Firefly luciferase* activity was normalized to galactosidase activity.

### Determination of mRNA levels

Total RNA was extracted from HepG2 cells using TRIzol (Invitrogen Life Technologies), quantified using a NanoDrop ND-1000 spectrophotometer (NanoDrop Technologies) and the RNA purity was determined by the A_260_/A_280_ ratio (all samples >1.8). Transcript levels were measured by quantitative real-time PCR. First-strand cDNA was synthesized from 1 μg RNA using the SuperScript III system (Invitrogen) and PCR analysis was conducted by the SYBR Green method on the ABI 7000 Sequence Detection System (Applied Biosystems). The relative amounts of mRNA were calculated from the Ct data applying calibration curves and normalized with hypoxanthine phosphoribosyltransferase (HRPT), TATA box binding protein (TBP) and hydroxymethylbilane synthase (HMBS) using GeNorm 3.5 software as described in [59]. Oligonucleotides used for qPCR are listed in Supplementary Table 1.

### Western blotting

The level of SND1, SREBP-2 and SREBP-1 protein was determined by Western blot analysis in the whole lysates and in the nuclear and cytoplasmic fractions of control and treated HepG2 cells. Cells were lysed and the nuclear and cytoplasmic fractions were separated using a Nuclear Extraction Kit (Panomics), according to the manufacturer's indications as described in [30]. Protein concentrations were determined using a commercially available kit (Bio-Rad). Ten micrograms of protein were loaded in each lane, fractionated on 9% SDS-PAGE at 170 V for 1 h and transferred to PVDF membranes (Bio-Rad) by semi-dry transference (1 h at 20 V). SND1, SREBP-2 and SREBP-1 were detected by immunoblotting using rabbit anti-SND1 [33], rabbit anti-SREBP-1 (H-160 Santa Cruz Biotechonolgy) and mouse anti-SREBP-2 (1C6 Santa Cruz Biotechonolgy) antibodies (0.3 μg/ml) respectively. Normalization was performed with β-tubulin (cytoplasm) or histone H3 (nucleus), using mouse anti-β-tubulin (Santa Cruz Biotechnology) and mouse anti-H3 (Cell Signaling Technology) primary antibodies. Horseradish peroxidase-conjugated goat anti-rabbit IgG (Sigma-Aldrich), and horse anti-mouse IgG (Sigma-Aldrich) were used as secondary antibodies. Detection was performed by ECL (GE Healthcare Life Sciences) and quantification by optical densitometry using QuantityOne software (Bio-Rad). Results are expressed as fold-change relative to the corresponding protein level in control cells.

### Electrophoretic mobility shift assays (EMSA)

Bicatenary DNA probes corresponding to SRE -60 (5’CAGGGTGCCTATTGGCCTGAGGGCCGGCGGG3’) and E-box -230 (5’GGCCAGATGCTGACGTGTCCTTTCCTTCC3’) sequences within SND1 proximal promoter were labelled with digoxigenin using the DIG Gel shift kit 2nd Generation (Roche Applied Science). For mobility shift assays, 1-2 μg nuclear extracts protein from HepG2 cells were incubated for 15 min at room temperature with 0.8 ng (0.04 pmol) of the labelled probes. For specific and non-specific competition assays, 100-fold molar excess of unlabelled probe was used. For supershift assays, 1 μg anti-SREBP-1 antibody or anti-SREBP-2 (Santa Cruz Biotechnology) or unspecific IgG was added. Electrophoresis, blotting, crosslinking and chemiluminescent detection were performed as described elsewhere [42].

### Chromatin immunoprecipitation (ChIP) assays

Chromatin Immunoprecipitation assays were performed using the EpiTectChIP One-Day Kit (SABiosciences) following manufacturer's instructions as described in [30]. The chromatin was immunoprecipitated with anti-SREBP-1 (H-160, Santa Cruz Biotechnology), anti-SREBP-2, (N-19 Santa Cruz Biotechnology) or non-immune serum IgG as negative control. DNA samples from immunoprecipitated (IP) material and from input were analysed by qPCR using the primers given in Supplementary Table 2. SYBR Green method was performed using 2-4 μl of the IP or input samples as templates and 0.1 μM specific primers. Results are given as the enrichment of the IP relative to the negative control and determined as 2^-(ΔΔCt)^ being ΔΔCt=(Ct_(IP)_-Ct_(input)_) – (Ct_(IgG)_-Ct_(input)_). Conventional PCR analyses were performed with 2 μl of immunoprecipitates or input samples and 0.4 μM specific primers under the following conditions: 94°C for 3 min; 35 cycles at 94°C for 20 sec, 59°C for 30 sec, and 72°C for 30 sec; and final extension at 72°C for 2 min. The samples were analysed on a 3% (w/v) agarose gel.

### Small interfering RNA (siRNA) gene silencing

For silencing endogenous SREBP-1 and SREBP-2, HepG2 cells were reverse transfected with 10 nM or 30 nM pre-designed siRNA against human SREBP-1 (Forward 505-523: CCACUCCAUUGAAGAUGUA, reverse 505-523: UACAUCUUCAAUGGAGUGG and forward 4044-4062: GGAGAGAGACGUGUACAUA, reverse 4044-4062: UAUGUACACGUCUCUCUCC) or SREBP-2 (Forward 1193-1211: CCCAUAAUAUCAUUGAGAA, reverse 1193-1211: UUCUCAAUGAUAUUAUGGG and forward 1566-1584: GGAUGAUGCAAAGGUCAAA, reverse 1566-1584: UUUGACCUUUGCAUCAUCC), or Silencer® negative control siRNA using Lipofectamine RNAiMAX transfection reagent according to the manufacturer´s instructions (Invitrogen Life Technologies). The knocking-down efficiency was checked by measuring target gene transcript by RT-qPCR and immunoblotting. After 48 or 72 h, cells were lysed with TRIzol for RNA analysis, or with lysis buffer for protein analysis. For SND1 transcriptional activity assay, after 48 h silencing, culture medium was replaced with fresh medium, cells were transfected for 24 h with SND1 gene promoter constructs and luciferase activity was measured as described above.

### Statistical analysis

The results are shown as the mean ± SD of at least 3 independent experiments, each performed in triplicate, except otherwise stated. Statistical significance of results was assessed by the unpaired Student's *t*-test using GraphPad Prism (version 5; GraphPad Software Inc., CA).

## SUPPLEMENTARY MATERIALS FIGURES AND TABLES



## Abbreviations

ChIP: chromatin immunoprecipitation
DMSO: dimethyl sulfoxide
EMSA: electrophoretic mobility shift assays
ER: endoplasmic reticulum
HMGCR: hydroxymethylglutaryl-coenzyme A reductase
IP: immunoprecipitated
LDLR: low density lipoprotein receptor
LPDS: lipoprotein deficient serum
LXR: liver X receptor
NF-Y: nuclear factor Y
SCAP: SREBP cleavage activating protein
SND1: staphylococcal nuclease domain-containing protein 1
Sp1: specificity protein 1
SREBP: sterol regulatory element (SRE) binding protein.
