# Supplementary material for: SREBP-2-driven transcriptional activation of human SND1 oncogene
Source: Oncotarget. 2017 Nov 21;8(64):108181–94. doi: 10.18632/oncotarget.22569 (PMC5746135; doi:10.18632/oncotarget.22569)
Supplement: Supplementary file 1 [file oncotarget-08-108181-s001.pdf]

SREBP-2-driven transcriptional activation of human SND1 oncogene

SUPPLEMENTARY MATERIALS

Cell proliferation assays

Cell proliferation was assessed by crystal violet staining (1). Cells (9,000 HepG2 or 5,000 HEK293 cells/well) were grown in 96 well plates and treated with siRNA as described in Materials and methods. After 8, 24, 48 or 72 h, cells were washed with phosphate buffered solution, fixed with 4% formaldehyde and stained with 0.25% crystal violet. After drying, 150 µl of 33% acetic acid was added to each well and the absorbance at 590 nm was measured in a Synergy™ HT Multi-Detection Microplate Reader (BioTek Instruments).

Cholesterol measurement

Lipids were thoroughly extracted from cells following the method of Bligh and Dyer (2), dried in a Savant SpeedVac concentrator (Thermo Scientific, Rockford, Il) and stored at -80ºC under N_2 until analysis. Concentrated extracts were dissolved in toluene, and the amount of free and esterified cholesterol was measured by optical densitometry after separation by thin layer chromatography as described previously (3).

REFERENCES

1. Gillies RJ, Didier N, Denton M. Determination of cell number in monolayer cultures. Anal Biochem. 1986; 159:109-113.
2. Bligh EG, Dyer WJ. A rapid method of total lipid extraction and purification. Can. J Biochem Physiol. 1959; 37:911-917.
3. Ruiz JI, Ochoa B. Quantification in the subnanomolar range of phospholipids and neutral lipids by monodimensional thin-layer chromatography and image analysis. J Lipid Res. 1997; 38:1482-1489.

```
-1332 taaacttaca atcttttaag agagcaaggg gccatgcact gtggttaatg catgtaatac
-1272 cagcacttta ggaggccgag gtaggagtat agcttgagcc caggagacca gcctgggcaa
-1212 catagtgaaa tctcagctct acaaaaaaaa aattagctgg gcgtggtagc gcggacccgt
-1152 ggtaccagct ccagctacac aggagactga ggcaggagga tcgcttgagc ctgggagtgt
-1092 caaggctgca gtgagccgtg atcgtgcccc cgcactccag cctgagcgac agagcgagac
-1032 tttgtctcaa aaaagccaca aaaccaaaga aacaaaaaga actgcaaggg aagtactgtc
 -972 ttcagtgtag agccaagttt tacgattaaa tgacataaca catgcaaggt ccttggttcc
 -912 acatagtggg cactcaacga atattagttc tcgctaaaca gtcacttaaa tatttgtagg
 -852 ccttggcttt cccgttcctt ccccgacctt tttactgggg ctagggaagg tatccggtgg
 -792 ctggaggcgc ttcacactgg gtcgggtgag agcgtgaagc gagccttttc cctaacgtca
 -732 cgggagcagc tgggaagaga ccgaagaacg agggtcactc tcccctgtat acgtggcatg
 -672 tccttcctct aagagacatt tattcctccc accaacaaac attaatggaa cgcctcacaa
 -612 gtgctagacc ccagcgacac aaacaagggc gaggcacact ctctgactac aaaaagggcg
 -552 cgcggccgcg tggcaggtca aagcccacat cggtaggagg ttccacaaag ctctgatttt
 -492 ccaagttctc tccatacttt tagccaaggt aaaatgtcgg aagtcgagtg gagcgggtac
 -432 ggagattcct ccaggactct gcctgcgtgg ctccaccctc ccaggccgca ttcccgggta
 -372 cagcggcgcc cacctaggtg ggtatacaag tgtgcgcccc tgaggccagc ggggaaggac
 -312 gtcatgggcg aggtcaggcg ggagggcgcg gctggctccg cccctggggc tcccatggcc
 -252 ccccgcgccc cgcggccaga tgctgacgtg tccttcctt cccactacac ctcccgacat
 -192 tacctactcc ggctgccccg gaagtcccgc cccatattta gcccatcctt gcaaatcaac
 -132 tctttcagcg cttcctggaa ctcaccaccg ttttggggac caagaggcgg ggctccgcga
 -72  gcagggtgcc tattggcctg agggccggcg ggctctgggc gctaattggg cggagggcgg
 -12  tggcgctctg gcacgcttgc gcggcgagta gaacgtgtgg cggcggcgga gatcgcgtct
```

SRE -60, E-box -230, SRE -772, E-box -934, SRE -1092; E-box -1237

Supplementary Figure 1: Partial nucleotide sequence of SND1 gene distal promoter [GenBank: EF690304]. The transcription start site (+1) is shown by an arrow, in bold. Boxes indicate predicted binding motives for SREBP transcription factors SRE (solid line) and E-box (dotted line). Two SRE motives are located at -772 and -1092 and two E-box elements at -934 and -1237.

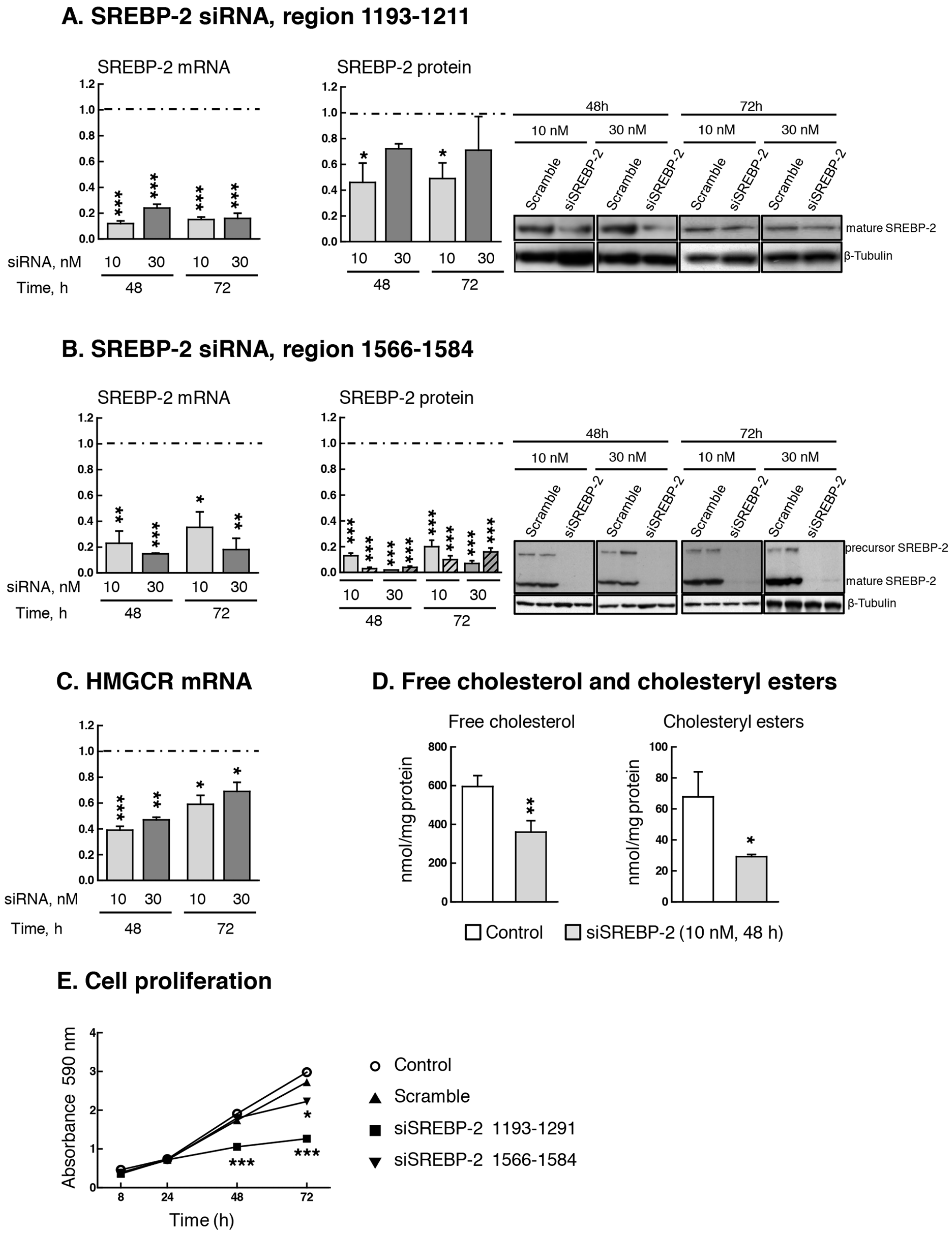

Supplementary Figure 2: Optimization of conditions for SREBP-2 silencing. HepG2 cells were reverse transfected with 10 nM (light grey) or 30 nM (dark grey) specific siRNAs against SREBP-2 transcript region 1193-1211 **(A)** or 1566-1584 **(B)**. After 48 or 72 h, RNA and protein were extracted and analyzed as described in Material and methods. Mature (plain bars) and precursor (striped bars) SREBP-2 were detected. HMGCR transcript **(C)** and cellular cholesterol levels **(D)** were reduced by SREBP-2 depletion. **(E)** Cell proliferation was determined by cristal violet assay. Levels in A-C are expressed relative to that in control cells, which is shown as a grid line. Results are reported as the mean ± SD of 3 independent experiments, each performed in duplicate (8 replicates in cell proliferation) and were analyzed by the two-tailed Student's-*t* test. Significance is denoted: * p≤0.05, ** p≤0.01 and *** p≤0.001 versus control cells.

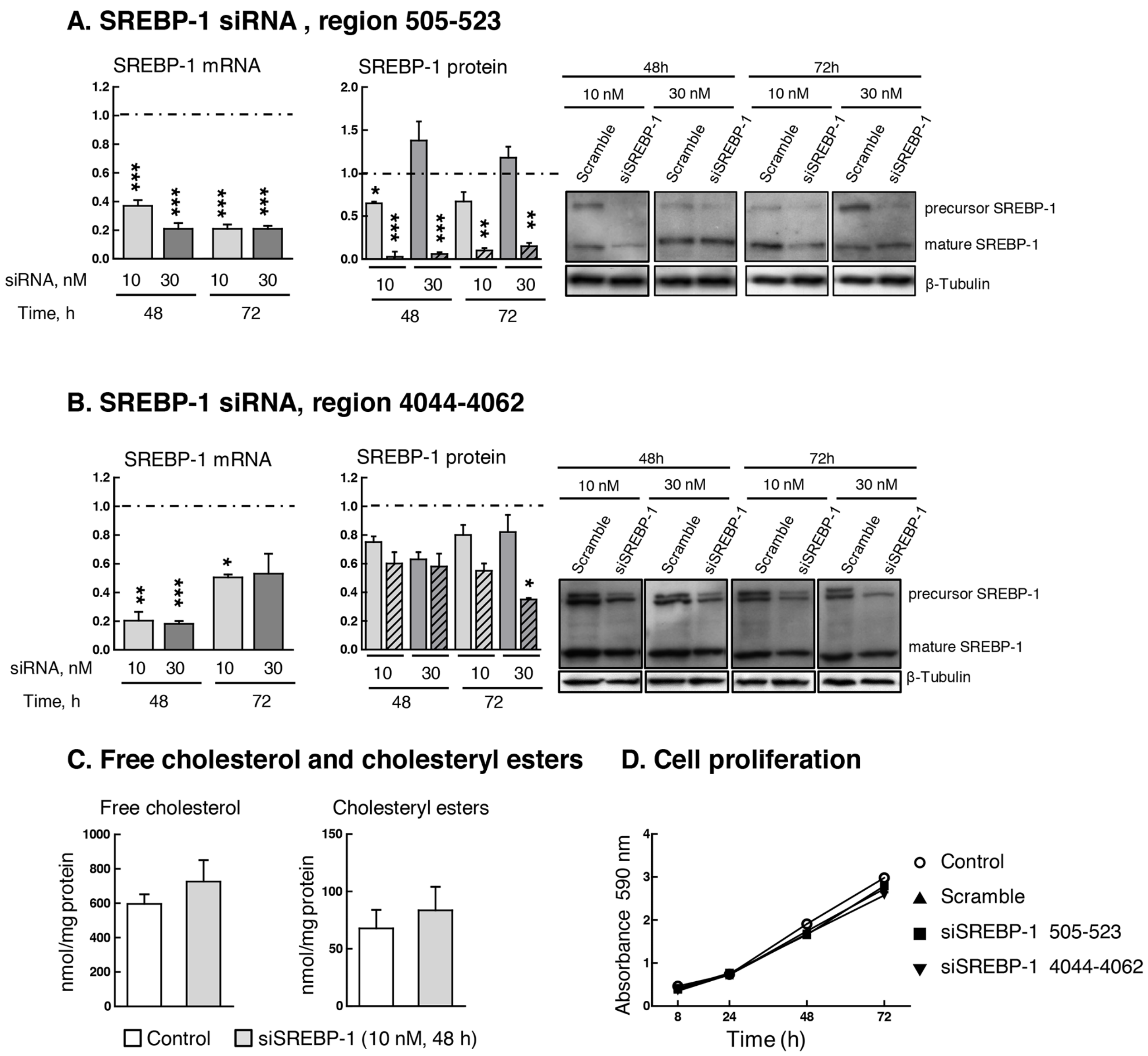

Supplementary Figure 3: Optimization of conditions for SREBP-1 silencing. HepG2 cells were reverse transfected with 10 nM (light grey) or 30 nM (dark grey) specific siRNAs against SREBP-1 transcript region 505-523 **(A)** or 4044-4062 **(B)**. After 48 or 72 h, RNA and protein were extracted and analyzed as described in Material and methods. Mature (plain bars) and precursor (striped bars) SREBP-1 were detected. **(C)** Cellular cholesterol levels were unaffected by SREBP-1 silencing. **(D)** Cell proliferation was determined by cristal violet assay. Levels in A-B are expressed relative to that in control cells, which is shown as a grid line. Results are reported as the mean ± SD of 3 independent experiments, each performed in duplicate (8 replicates in cell proliferation) and were analyzed by the two-tailed Student's-*t* test. Significance is denoted: * p≤0.05, ** p≤0.01 and *** p≤0.001 versus control cells.

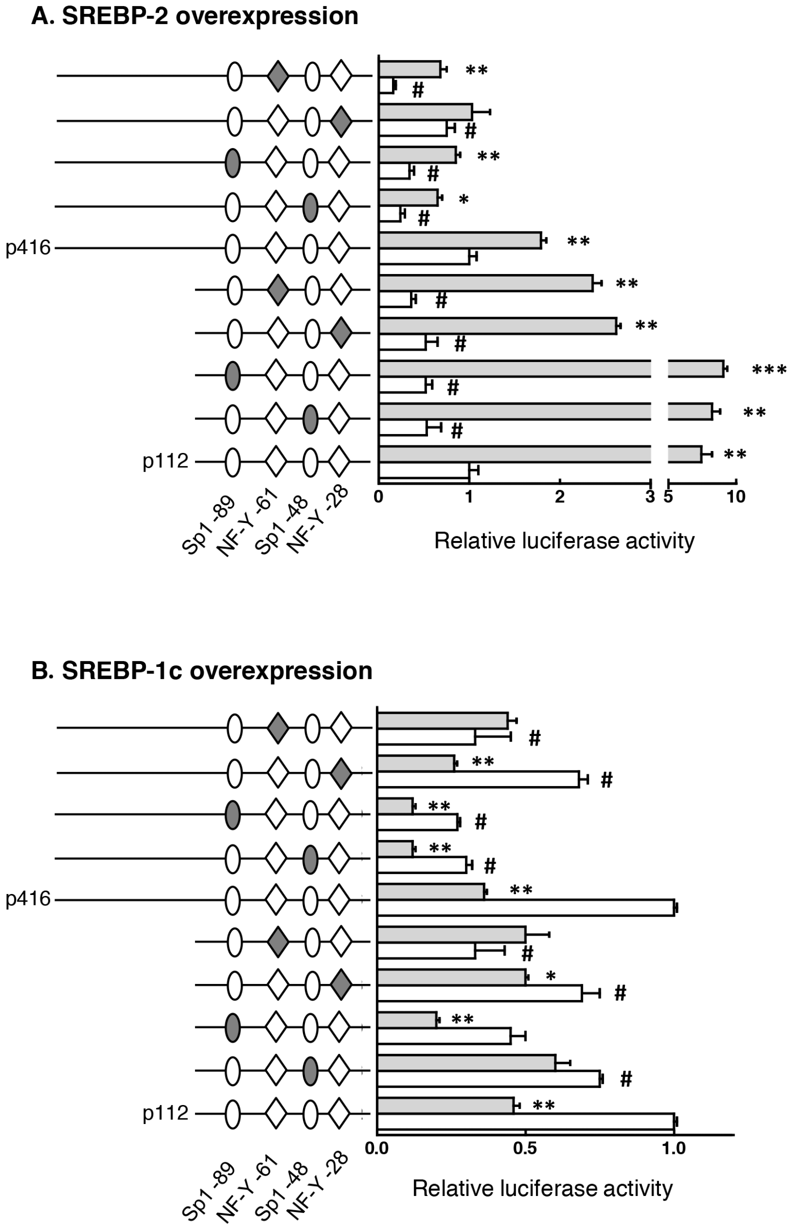

Supplementary Figure 4: Participation of NF-Y and Sp1 transcription factors in the SREBP-mediated control of SND1 promoter. HepG2 cells were cotransfected with SREBP-2 **(A)** or SREBP-1c **(B)** expression plasmids (dark bars) (or the corresponding control empty plasmid, white bars) and wild type or mutated SND1 promoter 5´deletion fragments p112 and p416 carrying single mutation (in grey) in the binding sites of NF-Y -28, Sp1 -48, NF-Y -61 or Sp1 -89, quite close to SRE -60. After 24 h, cells were harvested and luciferase activity measured as described in Material and methods. Promoter activity is represented as luciferase arbitrary units relative to its corresponding control, non-mutated p112 or p416 fragment. Results are reported as the mean ± SD of 3 independent experiments, each performed in quadruplicate and were analyzed by the two-tailed Student's-*t* test. * $p \leq 0.05$, ** $p \leq 0.01$ and *** $p \leq 0.001$ denote the effect of SREBP overexpression, # $p \leq 0.05$ denotes the effect of mutation.

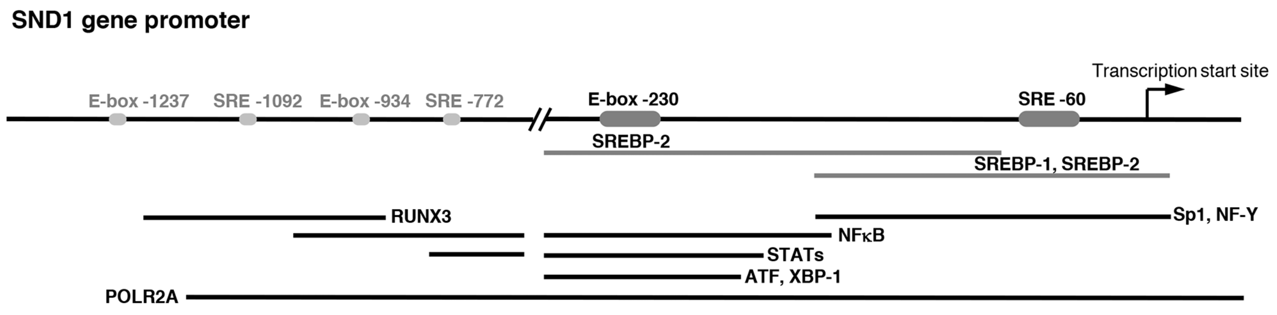

Supplementary Figure 5: Schematic representation of SND1 gene promoter indicating the location of the potential SREBP binding sites (light grey) and the regions validated by q-PCR (dark grey) from the anti-SREBP-1 or anti-SREBP-2 immunoprecipitated chromatin. Solid lines enclose the SND1 promoter occupancy by a set of transcription factors annotated in the UCSC Genome Browser (https://genome.ucsc.edu/).

Supplementary Table 1: Oligonucleotides used for mRNA quantification

Gene	Accession number		
SND1	NM_014390	Forward	GTGATCAGATACCGGCAGGATG
		Reverse	TCTTAATAGCTCTGGCCTCTGCAG
SREBP1	NM_001005291.2	Forward	ACAGCCATGAAGACAGACGG
		Reverse	ATAGGCAGCTTCTCCGCATC
SREBP2	NM_004599.2	Forward	ACAAGTCTGGCGTTCTGAGG
		Reverse	ACCAGACTGCCTAGGTCGAT
HMGCR	NM_000859.2	Forward	TTGGTGATGGGAGCTTGCTGTG
		Reverse	AGTCACAAGCACGTGGAAGACG
LDLR	NM_000527.4	Forward	TTCGAGTTCCACTGCCTAAG
		Reverse	TAACGCAGCCAACTTCATCG
HMBS	NM_000190.3	Forward	CACCCACACACAGCCTACTT
		Reverse	ACACTGTCCGTCTGTATGCG
TBP	NM_003194.3	Forward	TTGCAGTGACCCAGCAGCATCAC
		Reverse	AACCCTTGCGCTGGAACTCGTC
HPRT1	NM_000194.2	Forward	TTGCTTTCCTTGGTCAGGCA
		Reverse	ATCCAACACTTCGTGGGGTC

Supplementary Table 2: Oligonucleotides used for ChIP assays

SND1 (-268,-73).s	5’-CTGGGGCTCCCATGCCCC-3’
SND1 (-268,-73).as	5’-CGCGGAGCCCCGCCTCTT-3’
SND1 (-176,+4).s	5’-CCCGGAAGTCCCGCCCCATATT-3’
SND1 (-176,+4).as	5’-CGTTCTACTCGCCGCGCAAG-3’

Numbers indicate the amplicon in ChIP assays.
